# The Application of the Cameriere’s Methodologies for Dental Age Estimation in a Select KwaZulu-Natal Population of South Africa

**DOI:** 10.3390/dj10070130

**Published:** 2022-07-08

**Authors:** Sundika Ishwarkumar, Pamela Pillay, Manogari Chetty, Kapil Sewsaran Satyapal

**Affiliations:** 1 Department of Human Anatomy and Physiology, Faculty of Health Sciences, Doornfontein Campus, University of Johannesburg, Auckland Park P.O. Box 524, South Africa; sishwarkumar@uj.ac.za; 2 Department of Clinical Anatomy, School of Laboratory Medicine and Medical Sciences, College of Health Sciences, Westville Campus, University of KwaZulu-Natal, Private Bag X54001, Durban 4000, South Africa; satyapalk@ukzn.ac.za; 3 Department of Craniofacial Biology, Faculty of Dentistry, University of Western Cape, Private Bag X17, Bellville, Cape Town 7535, South Africa; mchetty@uwc.ac.za

**Keywords:** age estimation, Cameriere method, opened apices, panoramic radiographs, population-specific formulae, sex-specific formula

## Abstract

Background: The estimation of an individual’s age is a fundamental component of forensic odontology. Literary reports found that the efficiency of Cameriere methodology for age estimation varied among many population groups. Therefore, this study aimed to determine the applicability of the Cameriere methods to a select South African population of the KwaZulu-Natal (KZN) province. Materials and Methods: This cross-sectional retrospective study was conducted on 840 digital panoramic radiographs that met the inclusion criteria. Dental maturity was determined through the morphometric analysis of the seven left permanent mandibular and maxillary teeth in accordance with Cameriere et al. (2006). Moreover, the dental age was also calculated using the South African Black Bayesian formulae of the Cameriere method by Angelakopoulos et al. (2019). The paired sample *t*-test or Wilcoxon’s signed rank test assessed the significant difference between the chronological age and estimated dental age for the various formulae. A *p*-value < 0.05 was considered to be statistically significant. Results: The Cameriere et al. (2006) Italian formula and the South African Black Bayesian formulae of the Cameriere method by Angelakopoulos et al. (2019) underestimated and overestimated age in the South African Black and Indian population groups of the KZN province, respectively. Therefore, the authors generated a novel population-specific regression formulae (including and excluding third molars) using “step-wise regression analysis” and a “best-fit model” for the South African Black and Indian population groups of KZN. Conclusion: This study recommends that the population-specific formulae generated in this study be utilized in the KZN population to improve the accuracy of dental age estimation within this region.

## 1. Introduction

The estimation of an individual’s age is a fundamental component of forensic odontology, which is a branch of dentistry that examines dental evidence [1,2]. The demand for accurate age estimation is imperative for issues pertaining to illegal immigration (child labour), legal medicine (human trafficking/kidnapping), orthodontic treatment, criminal cases, adoption of individuals without birth certification and natural or mass disasters [2,3,4]. Furthermore, the South African “Child Justice Inter-Departmental Annual Report—2019/2020” stated that approximately 2984 individuals aged 17 years were awaiting trial within South Africa and in accordance with the South African Bill of Rights and Children’s Act—a “child” is an individual under the age of 18 years. Therefore, in cases where an individual is devoid of identification documentation, age estimation using non-invasive methods, such as dental panoramic radiographs, is often utilized to estimate age [5].

Furthermore, the assessment of dental characteristics is frequently utilized for the estimation of chronological age in children and adolescents. This is because the anatomical features on panoramic radiographs are the most reliable indicators of age estimation in the living [6,7]. Moreover, tooth formation is often utilized to assess dental maturity and estimate dental age and is preferred over tooth eruption, as tooth eruption is more often affected by environmental factors (i.e., nutrition, local trauma and mastication habits) than tooth formation (which is primarily controlled by genes) [7]. 

A comprehensive literature search was conducted on Google Scholar and PubMed using the key words “Cameriere method” and “Cameriere dental age”; the search was limited to studies conducted between 2006 and 2021. In 2006, Cameriere et al. used a software program to analyse panoramic radiographs in an Italian sample. An equation to describe the relationship between age and the morphometry of open apices in tooth roots of developing dentition for individuals aged between 5 and 15.99 years old was constructed [8,9]. In 2008, Cameriere et al. [10] validated the Cameriere’s method in European populations, as this method was reported to be more accurate than those of Demirjian et al. [11] and Willems et al. [12]. 

A number of studies have since validated the Cameriere methodology in their respective population groups, viz. Egyptian, Chinese, Brazilian, Malaysian, Indian, Turkish, Italian and Colombian [6,7,13,14,15,16,17]. El-Bakery et al. [6] and Fernandes et al. [13] reported that the Cameriere et al. [8] technique is accurate for the estimation of age in the Egyptian and Brazilian population groups, respectively, despite both studies reporting either an over and/or underestimation of age (Table 1). In accordance with literary reports the Cameriere et al. [8] method was recorded to underestimate age in the Malaysian population [18], Indian population [19], Turkish population [20], Turkish population [21] and Chinese population [7] (Table 1). Furthermore, Ozveren et al. [21] stated that the Cameriere et al. [8] method more significantly underestimated the dental age in females when compared to their male counterparts (Table 1). These population-specific differences may be ascribed to ancestry, environmental factors and variation in sample sizes or statistical analysis [4,22]. The efficiency of the Cameriere methodology for age estimation varied among many population groups and is not optimal for all population groups [20,23]. In addition, several studies have recommended the development of population-specific formulae [7,23,24].

KwaZulu-Natal is a coastal city located within South Africa, consisting of two main population groups, viz. South African Black (87%) and South African Indian (7%) [25]. To the best of the author’s knowledge, the applicability of the Cameriere [8] method (Italian formula) has not been evaluated, particularly in the South African Black and Indian population groups of KwaZulu-Natal (KZN). Only one study has employed the Cameriere European formula, established in 2007, on the South African Black and White population groups of the Gauteng province [26]. These authors reported that the Cameriere European formulae overestimated and underestimated age in younger and older South African (Black and White) children, respectively, therefore, they created population-specific formulae using the Bayesian calibration approach [26]. However, Yang et al. [7] stated that regional differences may exist within a country, therefore, it is imperative to create region-specific formulae in order to enhance the accuracy of the different dental age estimation methodologies in different regions. Moreover, it should be noted that the Black South African population consist of a number of sub-population groups, viz. Zulu, Xhosa, Sotho, Tswana, Venda, Ndebela, Swasi and Pedi. Therefore, sub-population differences may also exist due to genetic, climatic/environmental factors and dietary/nutritional differences [7]. Furthermore, to enhance the accuracy of age estimation, literary reports have suggested incorporating a combination of developing permanent dentition and third molars [7,27,28,29,30]. The aim of this study was to determine the applicability of the Cameriere [8] method (Italian formula) and the South African Black (female and male) Bayesian formulae of the Cameriere method [26] to the South African Black and Indian population groups of the KZN province.

## 2. Materials and Methods

### 2.1. Study Design and Sample

This cross-sectional retrospective study was conducted on 1300 digital panoramic radiographs aged between 5.00 to 25.99 years, obtained through consecutive sampling from private dental practitioners within the KZN province. Of the aforementioned radiographs, 840 digital panoramic radiographs met the inclusion criteria (South African Black population group = 420 and South African Indian population group = 420). The South African Black and South African Indian population groups are majority groups located within KwaZulu-Natal [25]. In this study, population groups were distinguished in accordance with “modern systems of racial classification”, which states that South Africa has four main population groups, viz. South African Black (origin in any of the native or African groups); South African Coloured, South African Indian (individuals of Asian descent) and South African White (individuals of European) descent [37,38]. The criteria used in the aforementioned racial classification scheme is skin colour and ancestry [38]. This age range was selected as the dentition undergoes various stages of development during this period, and in accordance with South African Census 2011, approximately 44% of South Africans is younger than 20 years [4,5,25]. All demographic data (i.e., date of birth, sex and population group) were captured from the patient records. The chronological age was calculated by subtracting the date of birth from the date the digital panoramic radiograph was captured. At the time of assessment, radiographs were numerically coded and de-identified of the above-mentioned demographic factors, thereby eliminating investigator bias. Radiographs were then categorized according to sex, population group and age (into yearly intervals) for statistical analysis and representation of results. There were ten radiographs per category (i.e., ten radiographs for South African Black females, aged 5.00 to 5.99). Each digital panoramic radiograph was analysed and measured utilizing the CS Imaging Software (Version: 7.0.20).

### 2.2. Ethics and Procedures

Ethical clearance was obtained (BE: 405/17) from the Biomedical Research Ethics Committee at the University of KwaZulu-Natal. This study received gate-keepers letter from the manager at the dental practices.

### 2.3. Selection Criteria

Radiographs obtained from patients with developmental anomalies or trauma or bones pathology associated with the maxilla and mandible, impacted, extracted or agenesis of dentition were excluded. Radiographs depicting positioning error or distortion due to movement were also not included. Furthermore, in this study, the South African White and South African Coloured population groups were excluded from the data analysis upon statistical advice due to an insufficient sample size. Any radiograph below 5.00 or above 25.99 or that had incomplete patient records were excluded from this study.

### 2.4. Radiographic Evaluation

#### 2.4.1. Cameriere Method: Italian Formula

Dental maturity was determined through the morphometric analysis of the 7 left permanent mandibular and maxillary teeth in accordance with Cameriere et al. [8]. This study utilized the left quadrant as no statistical difference between growth rate of dentition on right and side was documented in the literature [39]. Furthermore, Vadla et al. [2] concluded that the left side of panoramic radiographs showed “superior results” in comparison to the left side. Dental age was then estimated by employing Cameriere’s (Italian) linear Regression Formula [8]:

Age = 8.971 + 0.375g + 1.631(x_5_) + 0.674 (N_0_) − 1. 034s − 0.176s × N_0_.

- g = boys (1) and girls (0)
- x_5_ = $\frac{A5}{L5}$
- N_0_ = teeth with root development complete (i.e., apical completely closed)
- S = sum of the open apices (s = x_1_ + x_2_ + x_3_ + x_4_ + x_5_ + x_6_ + x_7_)
- Ai = radiographic distance between inner sides of the open apex, i.e., A_i_; i = 1…5
- For teeth with two roots, the sum of the distances between the inner sides of the two open apices, i.e., A_6_ = A_6_1 + A_6_2
- L_i_ = radiographic tooth length. (L_i_; I = 1…7)
- To prevent the effect of magnification and angulation difference of the panoramic radiographs, the measurement A_i_ will be by divided by the tooth length (L_i_), i.e., X_i_ = $\frac{\mathrm{Ai}}{\mathrm{Li}}$; i = 1…7)

#### 2.4.2. South African Black Bayesian Formulae of the Cameriere Method

The 7 left permanent maxillary and mandibular teeth were analysed and measured in accordance with the Cameriere et al. [8] method. Thereafter, dental age was calculated using the South African Black Bayesian formulae of the Cameriere method by Angelakopoulos et al. (2019) for males and females [26]:
Age = (S − β_0_) × (β_1_)^−1^if β_0_ + β_1_ × γ < S  = (S – β_0_ + β_2_ × γ) × (β_1_ + β_2_)^−1^if 0 < S ≤ β_0_ + β_1_ × γEstimatesBlack FemaleBlack Malesβ_0_ = 6.611β_0_ = 7.155β_1_ = −0.589β_1_ = −0.616β_1_ = −0.589β_2_ = 0.480γ = 10.5γ = 10.8

### 2.5. KZN Formulae of the Cameriere Method

If the Cameriere et al. [8] Italian formula and the South African Bayesian formulae of the Cameriere method by Angelakopoulos et al. [26] was not applicable to the selected sample, this study developed population-specific regression formulae using “step-wise regression analysis” and a “best-fit model” using R Statistical Computing Software of the R Core Team 2020. The “step-wise regression analysis” model provided the best coefficients for age prediction, as well as the associated estimates, standard error, t-value and *p*-value for each coefficient. The aforementioned coefficients was subsequently utilized in the regression formulae to predict dental age using the Cameriere method. The “step-wise regression analysis” model was conducted for both the left permanent maxillary and mandibular dentition (including and excluding third molars). Moreover, a “best-fit model” was used to determine which age range (lowest AIC values) was most suitable for age estimation. 

### 2.6. Intra-Observer and Inter-Observer Agreement

In order to standardize the method utilized, the intra- and inter-observer jointly assessed 10 radiographs to ensure reliability and reproducibility of this study. Each digital panoramic radiograph was analysed on the three different occasions (four weeks apart) by the first author using the CS Imaging Software to ensure intra-observer reliability. A second examiner evaluated 5% of the total sample (*n* = 42) utilizing the identical method to confirm inter-observer validity. The intra- and inter-observer error was calculated using the intraclass correlation coefficient test by comparing the two sets of data.

### 2.7. Statistical Analysis

The statistical analysis was conducted utilizing R Statistical Computing Software of the R Core Team 2020, PBC, Boston, MA, USA (R-version 3.6.3). Descriptive statistics included the mean values and range for each age interval. The paired sample *t*-test assessed the significant difference between chronological age and estimated dental age recorded using the Cameriere et al. [8] Italian formula, the South African Bayesian formulae of the Cameriere method by Angelakopoulos et al. [26] and the KZN Formulae of the Cameriere method. Moreover, the absolute mean error between the chronological age and estimated dental age was calculated. This study also determined if a correlation exists between the chronological age and estimated dental age using the Coefficient of Determination (R^2^). A *p*-value < 0.05 was considered to be statistically significant. 

## 3. Results

### 3.1. Cameriere Method: Italian Formula

The Cameriere et al. [8] Italian Formula underestimated the chronological age in the selected South African sample in both males and females (Table 2 and Table 3). Furthermore, the mean difference between the chronological age and the estimated dental age was smaller in the mandibular dentition than in the maxillary dentition (Table 2 and Table 3). In addition, a statistically significant difference was found between the chronological age and estimated dental age for both the South African Black female and male sample, as well as the South African Indian females and males sample (Table 2 and Table 3). A lower difference between the mean chronological age and mean dental age was recorded for males in comparison to females (Table 2 and Table 3).

### 3.2. South African Black Bayesian Formulae of the Cameriere Method

The South African Black Bayesian formulae of the Cameriere method by Angelakopoulos et al. (2019) [26] for females and males overestimated the chronological age for both the Black and Indian populations in KZN (Table 2 and Table 3). On the contrary, it underestimated age by 0.05 years for Indian males using the maxillary dentition (Table 2 and Table 3). A smaller difference between the mean chronological age and mean dental age was recorded in comparison to the Cameriere et al. [8] method, however, statistically significant differences were recorded between the aforementioned parameters. Only the South African Black females showed no statistically significant difference between the chronological age and dental age (*p*-value = 0.139) (Table 2 and Table 3). In contrast to the Cameriere et al. [8] Italian formula, the maxillary dentition generally had a smaller mean difference between the chronological and the mean dental age (Table 2 and Table 3). 

### 3.3. KZN Formulae of the Cameriere Method

The Cameriere et al. [8] Italian formula and the South African Bayesian formulae of the Cameriere method by Angelakopoulos et al. [26] did not apply to the selected South African population. Therefore, this study developed 16 regression formulae using “step-wise regression analysis” to predict dental age in the South African Black and Indian population groups of KZN (Table 4 and Table 5). Furthermore, the age ranges that yielded the best results (i.e., 5.00 to 15.99 and 5.00 to 19.99 years) were determined using a “best-fit model”, which to the best of our knowledge, was not done in previous studies. In addition, this study investigated and developed regression formulae for both the left permanent maxillary and mandibular dentition, while most studies only examined the seven left permanent mandibular dentition. This study also utilized the third molar dentition to determine age beyond 15.99 years and additional regression formulae were developed using the aforementioned method to include the third molar teeth. Therefore, Table 4 and Table 5 highlight the regression formulae based on the seven left maxillary and mandibular teeth (excluding third molars) and eight left maxillary and mandibular teeth (including third molars) for South African Black and Indian female and male individuals aged between 5.00 and 15.99 years and 5.00 and 19.99 years in KZN, respectively. 

The efficiency of the KZN formulae generated in this study were assessed on a further 60 digital panoramic radiographs that met the inclusion criteria using correlation coefficient analysis (R^2^) and paired sample *t*-test to determine the how closely the chronological age correlated with the estimated dental age. The mean difference for between the chronological age and estimated dental age using the KZN formulae for individuals aged between 5.00–15.99 years were 0.44 years and 0.29 years in the maxilla and mandible, respectively. The mean difference for individuals aged between 5.00–19.99 years using the KZN formulae were 0.51 years in the maxilla and 0.60 years in the mandible. No statistically significant difference was recorded between the chronological age and estimated dental age using the regression formulae generated in this study (*p*-value ≥ 0.05) (Table 6). Furthermore, excellent correlations between the chronological age and dental age were recorded using the regression formulae generated in this study (R^2^ > 0.9) (Table 6).

### 3.4. Intra-Observer and Inter-Observer Agreement

This study recorded an intra-observer agreement of 0.99 and inter-observer agreement of 0.97, which denotes an excellent agreement between the examinations.

## 4. Discussion

The accurate estimation of an individual’s age is imperative for forensic analysis, medico-legal issues (i.e., criminal prosecution and management of immigration issues) and anthropology, with osteology-based and dental-based methodologies being most frequently utilized for this purpose [40]. Tooth development is often used to assess dental maturity and estimate dental age, which is not only important for the fields of forensic odontology and forensic dentistry but also for clinical diagnosis and treatment in paediatric dentistry [41]. Furthermore, dental radiographs are valuable for forensic and archaeology purposes to estimate age in the living and dead [22]. Literary reports show that there is a global variation in dental maturation based on geographical and ancestry origins [22,42]. Limited studies have been conducted on the development of dentition in South African children, however, some studies have suggested that children of African ancestry have significantly advanced tooth formation and eruption profiles in comparison to children of European ancestry [26]. This discrepancy may be attributed to ancestry, geographical location and variation in sample sizes or statistical analysis [22]. 

The Cameriere et al. [8] Italian formula underestimated age in the select South African population groups of KZN, which concurred with the findings of previous studies conducted on other developing countries, viz. Egyptian and Indian [6,19,34,36]. Similarly, Cameriere et al. [43], Pinchi et al. [44]; Javadinejad et al. [4] and Rozylo et al. [35] reported an underestimation of age by 0.11 years, 0.96 years, 0.66 years and 0.18 years, respectively. Furthermore, Galic et al. [31] reported an underestimation of 0.02 years in males and overestimation by 0.09 years in females. On the contrary, Wolf et al. [22] reported that age was overestimated in 6 to 11 year old males and 6 to 10 year old females, but underestimated in 12 to 14 year old males and 11 to 14 year old females. However, De Luca et al. [3] and Javadinejad et al. [4] concluded that the Cmeriere method accurately estimated the dental age of Mexican and Iranian children. 

In the study of Angelakopoulos et al. [26], the Cameriere et al. [43] European formula underestimated age in younger children, while it overestimated age in older children within the South African Black and White population groups of the Gauteng province. Angelakopoulos et al. [26] then developed new population-specific formulae for the Black and White populations groups using the Bayesian calibration approach. This study validated the applicability of these formulae on the South African Black and Indian population groups of KZN, as literature has suggested that regional differences exists within the same country [7,36]. These formulae were also noted to overestimated age of the select population of KZN. Such differences in the rate of dental development within different regions were also reported by Altunsoy et al. [45] and Baylis and Bassed [46] in the Turkish and New Zealand population groups, respectively. Yang et al. [7] attributed these regional differences to genetics, socio-economic status, dietary and nutritional status, environmental factors and ancestry groups, as the authors further elaborated that “even in the same country, the dental development of different populations varies”.

In accordance with the recommendation of previous studies, specific formulae should be generated for different population and ancestry groups [7,47,48]. Therefore, this study generated population-specific formulae (excluding and including third molars) for the South African Black and Indian (female and male) population groups of KZN. The KZN Formulae of the Cameriere method revealed an excellent correlation between the chronological and dental ages for both models (including and excluding third molars), with R^2^ values greater than 0.9. In addition, a statistically insignificant correlation between the chronological age and estimated dental age using the KZN Formulae of the Cameriere method was recorded in this study. Furthermore, no statistically significant differences were recorded between the maxillary and mandibular formulae generated in this study for the two population groups. Therefore, results of this study were in agreement with those of Rai et al. [47], Alghali et al. [48] and Yang et al. [7] that conclude that population-specific and regional-specific norms generate more accurate and reliable age estimates than the Cameriere’s Italian formula and the South African Black Bayesian formulae of the Cameriere method.

## 5. Future Direction and Limitations 

This retrospective study was only able to access digital panoramic radiographs from Dental Practitioners located within urban areas of KZN, as these facilities are not readily available within rural areas. Furthermore, a number of the available panoramic radiographs were excluded from this study due to positioning errors and missing or impacted dentition or pathologies, which reduced the sample size. Furthermore, for statistical analysis, this study could only obtain the minimum number of scans for the South African Black and South African Indian population groups, which are majority groups located within KwaZulu-Natal [25]. In addition, non-essential exposure to radiation is not implemented in South Africa, therefore this study opted to utilise retrospective radiographs. This study recommends that the population-specific regression formulae generated in this study should be incorporated into future studies conducted in other regions of South Africa. In addition, the applicability of the Cameriere method should be tested on the South African Coloured population. 

## 6. Conclusions

The Cameriere et al. [8] Italian formula and the South African Black Bayesian formulae of the Cameriere method by Angelakopoulos et al. [26] underestimated and overestimated age in the South African Black and Indian population groups of KZN province, respectively. According to the literature reviewed, differences between the South African Black Bayesian formulae and the select KZN population groups may be attributed to regional and climate differences, socio-economic status and ancestry. Therefore, the authors generated population-specific regression formulae using “step-wise regression analysis” for the South African Black and Indian population groups of KZN to improve the accuracy of dental age estimation within this region.

## Figures and Tables

**Table 1:** Applicability of Cameriere et al. (2006) on other population groups.

Author	Year	Population	Sample Size	Age Range	Key Findings
El-Bakery et al. [6]	2009	Egyptian	286	5–16	Approximately 98% accurate for the estimation of age, however, age was underestimated by 0.43 years.
Galic et al. [31]	2011	Bosnian-Herzegovian	1089	6–13	Overestimated age by 0.09 years in girls and underestimated by 0.02 years in boys
Fernandes et al. [13]	2011	Brazilian	160	5–15	Reliable for age estimation—slight overestimation and underestimation were reported in the age categories 5–10 years and 11–15 years, respectively.
Bagh et al. [24]	2014	Indian	25	5–15	Slight overestimation but no statistical difference
Kumaresen et al. [18]	2014	Malaysian	426	5–15	Underestimation by 0.41 years but accurate for age estimation
Shrestha et al. [19]	2014	Indian	50	5–15	Underestimated by 0.11 years in boys and 0.23 years in girls
Gulsahi et al. [14]	2015	Turkish	603	8–15	Underestimation by 0.35 years
Javadinejab et al. [4]	2015	Iranian	577	3–15	Underestimated age by 0.19 years
Balla et al. [32]	2016	South Indian	150	7–14.99	Underestimated age
Wolf et al. [22]	2016	German	479	6–14	Males—Overestimation (6–11 years) and Underestimation (12–14 years) Females—Overestimation (6–10 years) and Underestimation (11–14 years)
Santana et al. [33]	2017	Mixed sample	360	6–17	Underestimated age in both males and females by -1.32 years and -1.19 years
Apaydin and Yasar [20]	2018	Turkish	330	5–15.90	Underestimation of age by 0.580 years
Nair et al. [34]	2018	Indian	10	7–12	Underestimation dental age
Rozylo et al. [35]	2018	Polish	2148	5–15	Underestimation dental age
Gannepalli et al. [36]	2019	Indian	200	10–15	Underestimated dental age by 1.50 years (male) and 1.54 years (females)
Ozveren et al. [21]	2019	Turkish	636	6–15	Underestimated age in both sexes
Yang et al. [7]	2021	Chinese	1803	4–22.99	Underestimation of age with a mean difference of 0.47 ± 1.11 years and 0.69 ± 1.19 years in males and females, respectively

**Table 2:** Mean Chronological and Dental age calculated using the Cameriere formulae for South African Black and Indian population group of KZN (in years).

Formula	Sample Size	Age Range	Sex	Population Group	Maxillary						Mandibular					
					Mean CA	Mean DA	Mean CA–DA	MAE	Correlation (R^2^)	*p*-Value	Mean CA	Mean DA	Mean CA–DA	MAE	Correlation (R^2^)	*p*-Value
Cameriere (2006) Italian [8]	440	5.00 – 15.99	B	SA Black & Indian	10.49	9.88	0.61	1.00	−0.68	<0.001	10.49	10.05	0.44	1.04	−0.67	<0.001
	220		F	SA Black & Indian	10.50	9.85	0.65	1.01	−0.68	<0.001	10.50	9.98	0.52	1.05	−0.66	<0.001
	220		M	SA Black & Indian	10.48	9.90	0.58	0.99	−0.68	<0.001	10.48	10.12	0.36	1.04	−0.68	<0.001
	220		B	SA Black	10.48	9.91	0.57	1.05	−0.66	<0.001	10.48	10.04	0.44	1.13	−0.65	<0.001
	110		F	SA Black	10.48	9.86	0.62	1.03	−0.67	<0.001	10.48	9.94	0.54	1.14	−0.67	<0.001
	110		M	SA Black	10.48	9.96	0.52	1.08	−0.64	<0.001	10.48	10.15	0.33	1.12	−0.65	0.006
	220		B	SA Indian	10.48	9.83	0.65	0.94	−0.70	<0.001	10.48	10.04	0.44	0.96	−0.69	<0.001
	110		F	SA Indian	10.53	9.85	0.68	0.99	−0.69	<0.001	10.53	10.02	0.51	0.96	−0.66	<0.001
	110		M	SA Indian	10.49	9.83	0.66	0.90	−0.71	<0.001	10.49	10.09	0.40	0.96	−0.72	<0.001
Bayesian SA Black Cameriere (2017) [26]	360	6.00 – 14.99	B	SA Black & Indian	10.49	10.65	−0.16	0.88	0.82	<0.001	10.49	10.74	−0.25	0.80	0.83	<0.001
	180		F	SA Black & Indian	10.50	10.68	−0.18	0.91	0.82	0.000	10.50	10.74	−0.24	0.83	0.83	<0.001
	180		M	SA Black & Indian	10.47	10.61	−0.14	0.89	0.82	0.000	10.47	10.73	−0.26	0.77	0.84	<0.001
	180		B	SA Black	10.46	10.71	−0.25	0.89	0.83	0.005	10.46	10.68	−0.22	0.87	0.83	<0.001
	90		F	SA Black	10.47	10.65	−0.18	0.84	0.83	0.139	10.47	10.63	−0.16	0.89	0.83	0.020
	90		M	SA Black	10.46	10.77	−0.31	0.94	0.82	0.006	10.46	10.72	−0.26	0.85	0.84	0.004
	180		B	SA Indian	10.52	10.58	−0.06	0.86	0.82	0.009	10.52	10.79	−0.27	0.72	0.83	<0.001
	90		F	SA Indian	10.53	10.71	−0.18	0.87	0.82	0.012	10.53	10.84	−0.31	0.75	0.83	0.001
	90		M	SA Indian	10.50	10.45	0.05	0.86	0.82	0.014	10.50	10.75	−0.25	0.70	0.83	0.003

B—both (male and female); F—female; M—male; SA—South African; CA—chronological age; DA—dental age; MEA—mean absolute error.

**Table 3:** Mean difference and absolute error using the Cameriere formulae for South African Black and Indian population group of KZN for each cohort (in years).

Age Cohorts (Year)	Sample Size (n)	South African Black Female				South African Black Male				South African Indian Female				South African Indian Male			
		Maxilla		Mandible		Maxilla		Mandible		Maxilla		Mandible		Maxilla		Mandible	
		MD	MAE	MD	MAE	MD	MAE	MD	MAE	MD	MAE	MD	MAE	MD	MAE	MD	MAE
Cameriere (2006) Italian Formula																	
5.00–5.99	40	−0.34	0.62	−0.90	0.96	−0.62	0.93	−1.33	1.33	−0.27	0.79	−1.00	1.04	0.06	0.51	−0.69	1.04
6.00–6.99	40	−0.37	0.58	−0.29	1.00	−0.54	0.66	−0.79	0.79	−0.02	0.71	−0.42	0.62	0.14	0.59	−0.53	0.60
7.00–7.99	40	0.05	0.33	−0.42	0.47	−0.58	0.67	−0.74	0.86	−0.04	0.28	−0.13	0.24	0.10	0.60	−0.34	0.56
8.00–8.99	40	−0.51	0.97	−0.57	0.86	0.02	0.29	−0.09	0.80	0.63	1.12	0.38	0.78	0.17	0.44	−0.31	0.59
9.00–9.99	40	0.42	0.64	0.09	0.48	−0.19	0.68	−0.18	0.73	0.23	0.74	0.07	0.81	0.36	0.55	−0.11	0.63
10.99–10.99	40	0.49	0.72	0.38	0.60	0.48	0.94	0.12	0.74	0.31	0.58	0.12	0.34	0.18	0.96	−0.08	0.71
11.99–11.99	40	0.39	0.78	0.36	0.78	0.30	0.83	0.22	0.72	0.80	0.80	0.80	0.80	0.49	0.53	0.56	0.56
12.99–12.99	40	1.51	1.51	1.37	1.40	0.97	1.05	1.26	1.26	1.30	1.33	1.27	1.30	0.86	0.86	0.96	0.96
13.99–13.99	40	1.36	1.40	1.76	1.82	1.48	1.48	1.41	1.41	0.65	0.65	0.67	0.79	1.13	1.22	1.12	1.18
14.99–14.99	40	1.60	1.60	1.63	1.63	2.30	2.29	1.84	1.84	1.59	1.59	1.48	1.48	1.41	1.41	1.50	1.50
15.99–15.99	40	2.26	2.26	2.59	2.59	2.06	2.06	1.85	1.85	2.26	2.26	2.34	2.34	2.27	2.27	2.27	2.27
Bayesian SA Black Cameriere (2017) Formula																	
6.00–6.99	40	0.48	1.15	0.98	1.72	0.38	1.17	−0.01	1.02	0.79	1.23	0.02	0.89	1.57	1.78	0.21	0.61
7.00–7.99	40	0.01	0.97	−0.58	0.85	−0.85	1.20	−1.07	1.15	−0.17	0.53	−0.46	0.57	0.35	1.38	−0.50	1.02
8.00–8.99	40	−1.33	1.33	−1.21	1.22	−0.81	1.11	−0.27	1.53	0.32	0.78	−0.14	0.57	−0.36	0.75	−0.85	0.90
9.00–9.99	40	−0.02	0.64	−0.19	0.36	−1.03	1.03	−0.81	0.93	−0.16	0.60	−0.04	0.80	−0.49	0.65	−0.51	0.77
10.99–10.99	40	−0.53	0.81	−0.40	0.86	−0.12	0.68	−0.23	0.53	−0.61	0.96	−0.73	0.94	0.03	0.63	−0.06	0.47
11.99–11.99	40	−0.41	0.94	−0.51	0.70	−0.92	0.99	−0.60	0.81	−0.87	1.50	−0.54	1.12	−0.59	1.02	−0.54	0.96
12.99–12.99	40	−0.32	0.71	−0.29	1.11	−0.27	0.71	0.15	0.58	−0.85	0.91	−0.68	0.85	−0.45	0.61	−0.33	0.67
13.99–13.99	40	−0.04	0.44	0.03	0.49	0.13	0.79	−0.21	0.40	−0.61	0.64	−0.53	0.55	−0.10	0.44	−0.15	0.37
14.99–14.99	40	0.51	0.61	0.65	0.73	0.73	0.73	0.70	0.73	0.56	0.65	0.38	0.47	0.50	0.50	0.49	0.49

MD—mean difference (CA-DA)—a negative value indicates overestimation and a positive value indicated underestimation; MAE—mean absolute error.

**Table 4:** Estimation of chronological age using step-wise regression analysis for South African Black and Indian population groups of KZN in individuals aged between 5.00 and 15.99 years (excluding third molars).

Maxillary					Mandibular				
Coefficients	Estimates	Standard Error	*t*-Value	*p*-Value	Coefficients	Estimates	Standard Error	*t*-Value	*p*-Value
South African Black Females (KZN)									
Age = 10.06 − 4.14(X_1_) -1.59(X_5_) -1.78(X_7_) + 0.66(N_0_)					Age = 10.50 – 1.00(s) + 0.59(N_0_) + 7.66(X_1_) – 4.30(X_4_)				
Intercept	10.06	0.33	30.14	<0.001	Intercept	10.50	0.41	25.83	<0.001
Max X_1_	−4.14	1.65	−2.50	0.013	S	−1.00	0.53	−1.89	0.061
Max X_5_	−1.59	0.77	−2.07	0.041	N_0_	0.59	0.09	6.73	<0.001
Max X_7_	−1.78	0.47	−3.75	0.0003	Man X_1_	7.66	2.31	3.32	0.001
N_0_	0.66	0.07	9.34	<0.001	Man X_4_	−4.30	2.93	−1.47	0.146
South African Black Males (KZN)									
Age = 9.70 – 5.20(X_3_) – 0.89 (X_7_) + 0.84 (N_0_)					Age = 9.68 – 1.30(s) + 0.81(N_0_) + 4.33(X_6_)				
Intercept	9.70	0.29	32.72	<0.001	Intercept	9.68	0.36	27.10	<0.001
Max X_3_	−5.20	1.08	−4.79	<0.001	S	−1.30	0.20	−6.50	<0.001
Max X_7_	−0.89	0.31	−2.84	0.005	N_0_	0.81	0.08	10.68	<0.001
N_0_	0.84	0.07	12.64	<0.001	Man X_6_	4.33	1.50	2.88	0.005
South African Indian Female (KZN)									
Age = 10.47 + 2.73(X_2_) – 2.65(X_3_) – 3.99(X_5_) – 6.81(X_6_) – 0.64(X_7_) + 0.58 (N_0_)					Age = 9.91 – 1.23(s) + 0.68(N_0_)				
Intercept	10.47	0.34	30.64	<0.001	Intercept	9.91	0.28	35.79	<0.001
Max X_2_	2.73	1.01	2.69	<0.001	S	-1.23	0.11	-10.83	<0.001
Max X_3_	−2.65	1.69	−1.56	0.122	N_0_	0.68	0.06	12.09	<0.001
Max X_5_	−3.99	1.12	−3.57	<0.001					
Max X_6_	−6.81	2.61	−2.61	0.01					
Max X_7_	−0.64	0.33	−1.96	0.052					
N_0_	0.58	0.07	8.92	<0.001					
South African Indian Male (KZN)									
Age = 10.71 + 5.06(X_2_) – 2.80(X_4_) – 1.82(X_5_) – 3.76(X_6_) – 1.79(X_7_) + 0.59(N_0_)					Age = 10.43 – 2.30(s) + 0.64(N_0_) + 4.99(X_2_) + 4.37(X_3_) + 3.03(X_6_)				
Intercept	10.71	0.30	36.28	<0.001	Intercept	10.43	0.39	26.74	<0.001
Max X_2_	5.06	1.53	3.30	0.001	S	−2.30	0.36	−6.42	<0.001
Max X_4_	−2.80	1.10	−2.56	<0.001	N_0_	0.64	0.08	8.54	<0.001
Max X_5_	−1.82	0.86	−2.12	0.037	Man X_2_	4.99	2.51	1.98	0.050
Max X_6_	−3.76	0.94	−4.01	<0.001	Man X_3_	4.37	1.86	2.35	0.028
Max X_7_	−1.79	0.32	−5.59	<0.001	Man X_6_	3.03	1.17	2.58	0.011
N_0_	0.59	0.06	9.79	<0.001					

**Table 5:** Estimation of chronological age using step-wise regression analysis for South African Black and Indian population groups of KZN in individuals aged between 5.00 and 19.99 years (including third molars).

Maxillary					Mandibular				
Coefficients	Estimates	Standard Error	*t*-Value	*p*-Value	Coefficients	Estimates	Standard Error	*t*-Value	*p*-Value
South African Black Females (KZN)									
Age = 9.45 − 3.79(X_3_) − 1.76(X_7_) + 1.06(N_0_)					Age = 9.77 − 1.49(s) + 1.03(N_0_) − 0.27(X_8_) + 8.12(X_1_)				
Intercept	9.45	0.46	20.65	<0.001	Intercept	9.77	0.61	15.97	<0.001
Max X_3_	−3.79	1.79	−2.12	0.036	S	−1.49	0.29	−5.08	<0.001
Max X_7_	−1.76	0.63	−2.77	0.006	N_0_	1.03	0.10	10.32	<0.001
N_0_	1.06	0.08	13.19	<0.001	Man X_8_	−0.27	0.17	−1.59	0.115
					Man X_1_	8.12	2.80	2.90	0.004
South African Black Males (KZN)									
Age = 10.47 − 6.92(X_3_) − 0.99(X_7_) + 0.97(N_0_) − 0.36(X_8_)					Age = 11.10 − 1.98(s) + 0.87(N_0_) − 0.80(X_8_) + 7.80(X_6_)				
Intercept	10.47	0.43	24.40	<0.001	Intercept	11.10	0.54	20.58	<0.001
Max X_3_	−6.92	1.56	−4.42	<0.001	S	−1.98	0.29	−6.85	<0.001
Max X_7_	−0.99	0.47	−2.13	0.034	N_0_	0.87	0.09	10.12	<0.001
N_0_	0.97	0.07	13.06	<0.001	Man X_8_	−0.80	0.22	−3.58	<0.001
Max X_8_	−0.36	0.14	−2.49	0.014	Man X_6_	7.80	2.26	3.46	<0.001
South African Indian Female (KZN)									
Age = 13.46 + 11.70(X_1_) + 3.31(X_2_) − 9.72(X_3_) − 7.92(X_5_) − 8.19(X_6_) − 1.35(X_7_) + 0.45(N_0_) − 0.88 (X_8_)					Age = 13.15 − 2.76(s) + 0.54(N_0_) − 1.59(X_8_) + 7.92(X_2_) − 6.72(X_3_) + 12.40(X_6_)				
Intercept	13.46	0.57	23.59	<0.001	Intercept	13.15	0.54	24.29	<0.001
Max X_1_	11.70	5.45	2.15	0.033	S	−2.76	0.63	−4.36	<0.001
Max X_2_	3.31	2.13	1.56	0.121	N_0_	0.54	0.08	6.35	<0.001
Max X_3_	−69.72	3.59	−2.71	0.008	Man X_8_	−1.59	0.31	−5.06	<0.001
Max X_5_	−7.92	2.02	−3.92	<0.001	Man X_2_	7.92	4.40	1.80	0.074
Max X_6_	−8.19	5.04	−1.63	0.106	Man X_3_	−6.72	4.55	−1.48	0.141
Max X_7_	−1.35	0.59	−2.30	0.023	Man X_6_	12.40	5.59	2.22	0.028
N_0_	0.45	0.09	4.93	<0.001					
Max X_8_	−0.88	0.19	−4.72	<0.001					
South African Indian Male (KZN)									
Age = 10.17 + 5.50(X_2_) − 2.30(X_3_) − 2.71(X_4_) − 2.86(X_6_) − 1.86(X_7_) + 0.97(N_0_) − 0.39(X_8_)					Age = 9.44 − 1.31(s) + 1.09(N_0_) − 0.46(X_8_) + 8.89(X_1_)				
Intercept	10.17	0.43	23.50	<0.001	Intercept	9.44	0.44	21.66	<0.001
Max X_2_	5.50	2.26	2.44	0.016	S	−1.31	0.25	−5.17	<0.001
Max X_3_	−2.30	1.52	−1.51	0.132	N_0_	1.09	0.07	15.68	<0.001
Max X_4_	−2.71	1.46	−1.85	0.066	Man X_8_	−0.46	0.22	−2.09	0.038
Max X_6_	−2.86	1.29	−2.22	0.028	Man X_1_	8.89	3.79	2.35	0.020
Max X_7_	−1.86	0.46	−4.01	<0.001					
N_0_	0.97	0.07	14.08	<0.001					
Max X_8_	−0.39	0.14	−2.74	<0.001					

**Table 6:** Efficiency of the KZN Formulae of the Cameriere method.

Formulae	Age Range	Correlation (R^2^)	*p*-Value
Maxillary			
Cameriere KZN Black Female (Excluding M3)	5.00–15.99	0.92	0.2
Cameriere KZN Black Female (Including M3)	5.00–19.99	0.92	0.5
Mandibular			
Cameriere KZN Black Female (Excluding M3)	5.00–15.99	0.91	0.3
Cameriere KZN Black Female (Including M3)	5.00–19.99	0.92	0.5
Maxillary			
Cameriere KZN Black Male (Excluding M3)	5.00–15.99	0.94	0.2
Cameriere KZN Black Male (Including M3)	5.00–19.99	0.93	0.1
Mandibular			
Cameriere KZN Black Male (Excluding M3)	5.00–15.99	0.94	0.2
Cameriere KZN Black Male (Including M3)	5.00–19.99	0.93	0.3
Maxillary			
Cameriere KZN Indian Female (Excluding M3)	5.00–15.99	0.95	0.5
Cameriere KZN Indian Female (Including M3)	5.00–19.99	0.91	0.2
Mandibular			
Cameriere KZN Indian Female (Excluding M3)	5.00–15.99	0.95	0.5
Cameriere KZN Indian Female (Including M3)	5.00–19.99	0.90	0.2
Maxillary			
Cameriere KZN Indian Male (Excluding M3)	5.00–15.99	0.96	0.7
Cameriere KZN Indian Male (Including M3)	5.00–19.99	0.95	0.4
Mandibular			
Cameriere KZN Indian Male (Excluding M3)	5.00–15.99	0.95	0.7
Cameriere KZN Indian Male (Including M3)	5.00–19.99	0.95	0.4

## Data Availability

Not applicable.
